# Auditory Cortical and Hippocampal-System Mismatch Responses to Duration Deviants in Urethane-Anesthetized Rats

**DOI:** 10.1371/journal.pone.0054624

**Published:** 2013-01-23

**Authors:** Timo Ruusuvirta, Arto Lipponen, Eeva Pellinen, Markku Penttonen, Piia Astikainen

**Affiliations:** 1 Department of Psychology, University of Turku, Turku, Finland; 2 Centre for Learning Research, University of Turku, Turku, Finland; 3 Department of Neurobiology, AIV Institute, Faculty of Health Sciences, University of Eastern Finland, Kuopio, Finland; 4 Department of Psychology, University of Jyväskylä, Jyväskylä, Finland; University of Salamanca- Institute for Neuroscience of Castille and Leon and Medical School, Spain

## Abstract

Any change in the invariant aspects of the auditory environment is of potential importance. The human brain preattentively or automatically detects such changes. The mismatch negativity (MMN) of event-related potentials (ERPs) reflects this initial stage of auditory change detection. The origin of MMN is held to be cortical. The hippocampus is associated with a later generated P3a of ERPs reflecting involuntarily attention switches towards auditory changes that are high in magnitude. The evidence for this cortico-hippocampal dichotomy is scarce, however. To shed further light on this issue, auditory cortical and hippocampal-system (CA1, dentate gyrus, subiculum) local-field potentials were recorded in urethane-anesthetized rats. A rare tone in duration (deviant) was interspersed with a repeated tone (standard). Two standard-to-standard (SSI) and standard-to-deviant (SDI) intervals (200 ms vs. 500 ms) were applied in different combinations to vary the observability of responses resembling MMN (mismatch responses). Mismatch responses were observed at 51.5–89 ms with the 500-ms SSI coupled with the 200-ms SDI but not with the three remaining combinations. Most importantly, the responses appeared in both the auditory-cortical and hippocampal locations. The findings suggest that the hippocampus may play a role in (cortical) manifestation of MMN.

## Introduction

Any changes in the invariant attributes of the auditory environment are of potential importance for survival. The rapid, effortless, and sensitive detection of auditory changes is, therefore, of crucial importance for the organism.

In humans, the preattentive detection of auditory changes is indexed by an electrical brain response with a fronto-central scalp distribution termed the mismatch negativity (MMN) of event-related potentials (ERPs) [1]–[3]. MMN can be elicited in the latency range of 150–250 ms even by changes perceptually too weak to attract involuntary attention [1]. If the auditory changes are of high magnitude, they can capture involuntary attention [4] and elicit a partly hippocampally-contributed P3a of ERPs in the later latency range of 250–280 ms [1], [5], [6].

MMN is typically experimentally addressed by using the so-called oddball condition. In this condition, a (‘deviant’) tone rarely replaces a frequently repeated (‘standard’) tone. MMN is elicited by the deviant tone only if the distinguishing feature(s) of the standard relative to the deviant are successfully represented neurally, stored in transient auditory memory, and compared to the auditory input by the deviant [1], [7], [8]. MMN is quantified by arithmetically subtracting ERPs to the standard tone from ERPs to the deviant tone. The specificity of MMN to deviant tones as changes in standard tones is reflected by the disappearance of these responses by the removal of standard tones from the series [3].

Mismatch responses (i.e., higher-amplitude brain responses to deviant tones than standard tones irrespectively of the polarity of these responses and despite these tones are only passively listened to) have been found in sleeping infants [9]–[11] and in awake, sleeping and anesthetized animals [12]–[23] (for negative findings in anesthetized rats, see, [24], [25]).

The generators of MMN are largely unknown. Different and partially contradictory explanations have been proposed [8], [26]–[28]. The general consensus points to the generation of MMN in the temporal and, subsequently, frontal cortical areas [29], [30] with a possible contribution from lower levels of the auditory pathway [31], [32].

No role in human MMN generation has been assigned to the hippocampus. Studies employing intracranial hippocampal recordings in neurological patients have reported no mismatch responses [33] or other, longer-latency differential responses to deviant tones as novel or salient stimuli rather than as changes in standards [34].

The MMN-hippocampus dissociation may, however, be premature for a number of reasons. First, too few hippocampal MMN data have been gathered in humans. Second, the hippocampus has been proposed, comparably to MMN [1], to respond by itself to rare stimuli on the basis of their discrepancy from a neural model of past repetitive stimuli [35]–[38]. Finally, hippocampal mismatch responses have been observed in animals [14], [16], and some of these responses have, comparably to MMN [3], even shown specificity to deviant tones as changes in standard tones [16].

Nevertheless, hippocampal mismatch responses could be claimed to be secondary to and solely triggered by auditory cortical mismatch responses in a limited set of circumstances. Namely, these responses could be argued to only emerge to high-magnitude deviant tones that capture involuntary attention in contrast to auditory cortical mismatch responses also observable to low-magnitude deviant tones close to their behavioral detection threshold. This would be analogous to a MMN-P3a sequence in humans that is only observable in full (i.e., also including P3a) when deviant tones of high magnitude attract involuntary switches of attention [1], [4].

In awake and anesthetized rats, cortical mismatch responses have been found to occur in the latency range of 30–150 ms [18], [19], [21]–[23] (for 25-ms onset latency, see [20]). In urethane anesthetized rats, auditory cortical mismatch responses have, comparable to MMN [1], been observed to be specific to deviant tones as changes per se in standard tones. That is, mismatch responses in these animals have been found to disappear when standard tones have either been completely removed from the series [18] or replaced by a set of heterogeneous tones of equal rarity to deviant tones [19]. Awake rats also show P3a-type responses to deviant tones of high magnitude. Similarly to P3a in humans [4], [6], these responses follow mismatch responses at about 240 ms post-stimulus [39].

**Figure 1:**
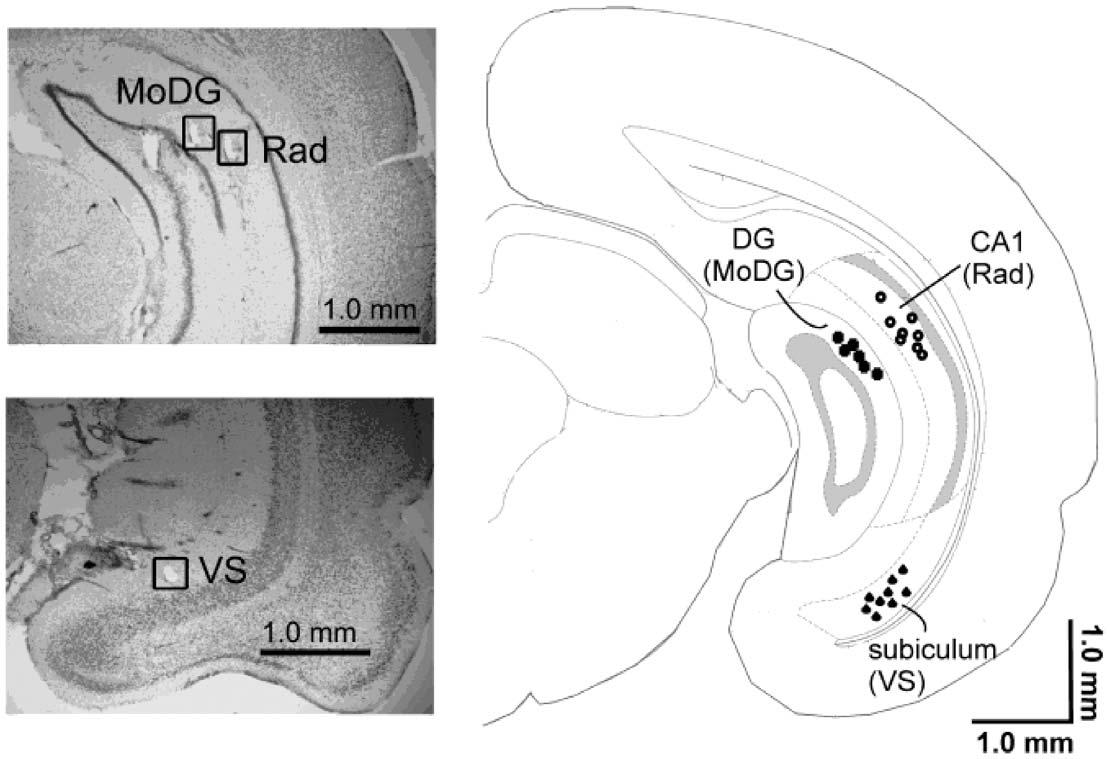
Electrode locations. The representative histological cresyl violet stained sections and a drawing of a coronal section of a rat brain 6.48 mm from bregma illustrate the locations of electrodes in the CA1 radiatum layer (Rad), in the molecular layer of the dentate gyrus (MoDG) and in the ventral subiculum (VS). Adapted from [54].

**Figure 2:**
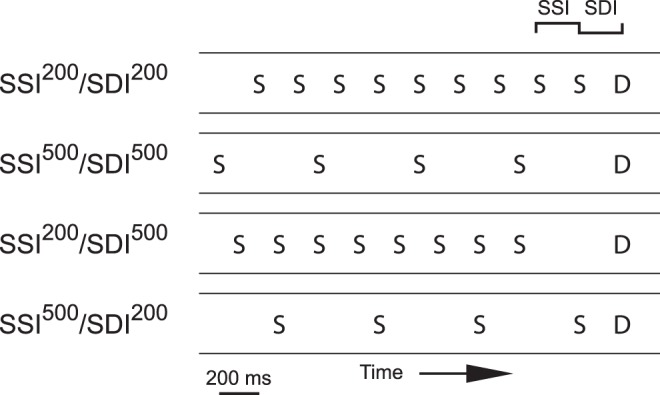
Experimental stimuli. In each of the four stimulus conditions, a duration deviant tone (D) was interspersed with a repeated standard tone (S). In the SSI^200^/SDI^200^ and SSI^500^/SDI^500^-conditions, the duration of the deviant was 25 ms of the standard 75 ms. In the SSI^200^/SDI^500^ and SSI^500^/SDI^200^-conditions, each duration was assigned to each stimulus type in separate series. Otherwise, the four conditions differed in stimulus-onset-asynchrony (200 ms or 500 ms) between consecutive standards (SSI) and between deviants and their immediately preceding standards (SDI).

In the present study, local-field potentials (LFPs) in urethane-anesthetized rats were recorded on the surface of the primary auditory cortex and in three locations of the hippocampal system (dentate gyrus, CA1, and subiculum, Figure 1). The recordings were made during four oddball conditions with different stimulus-onset asynchronies between consecutive standard tones and between deviants tones and standard tones immediately preceding them adapted from [40] (Figure 2). The deviant tone differed from the standard tone in duration (25 vs. 75 ms). Sound frequency was not used due to its spatial coding from the cochlea [41] owing to a potential confounding effect of lower levels of refractoriness in afferent pathways activated by the rare deviant tone than the frequently occurring standard tones [1]. Early neuronal selectivity to duration in the auditory system (the auditory midbrain) has been demonstrated but through active rather than fatigue-type of passive neural events [42]. We expected that some of the auditory oddball conditions would be more favorable for mismatch responses than others. Our interest was whether mismatch responses, whenever elicited, occupied both the auditory cortex and the hippocampus in a typical latency range of mismatch responses in urethane anesthetized rats or whether mismatch responses were only manifested in the auditory cortex and possibly only followed by later hippocampal activity.

## Results

We averaged the LFPs for each stimulus type (the deviant tone and the standard tone which immediately preceded the deviant tone) in each brain location, stimulus condition (Figure 2), and animal. From these averaged LFP waveforms, the mean amplitudes of three consecutive post-stimulus 37.5-ms time segments were calculated from 51.5 ms post-change (76.5 ms post-stimulus) onwards (see also [18], [19]).

### Auditory Cortex

**Figure 3:**
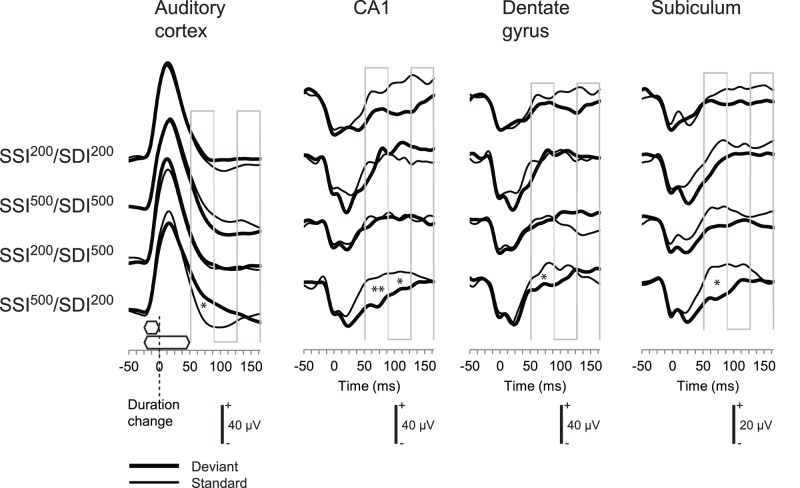
Electrophysiological results. LFPs in response to different stimulus types in different stimulus conditions. The zero time point is at the moment of change, that is, at 25 ms from tone onset and, thus, at the offset of the 25-ms tone. The two tones used as the deviant and the standard are illustrated above the x-axis of the panel for the auditory cortex. The three consecutive time segments for calculating average response amplitudes are illustrated in each panel. Significant differences in response amplitude between the deviant and the standard are marked between the curves. * *P*<0.05 and **P<0.01 in post-hoc uncorrected comparisons (paired t-tests).

In the auditory cortex, both stimulus types in all conditions could be seen to evoke a large positive deflection peaking at about 40 ms from stimulus onset (i.e., not from duration deviance). This deflection gradually sloped back to the baseline level, or below, by about 100 ms from stimulus onset (Figure 3).

The LFPs were displaced towards positive polarity in response to the deviant tone relative to the standard tone (Figure 3), as reflected by a stimulus (deviant tone, standard tone)×time-segment (first, second, third) -interaction, in the SSI^500/^SDI^200^ condition, F(2,18) = 5.13, P = 0.048. Post-hoc pairwise comparisons further indicated that this displacement was present (P<0.05) in the first time-segment (51.5–89 ms from duration change). In the other conditions, no significant stimulus×time-segment interactions were observed (P≥0.532).

No main effect of stimulus was present in any condition (P≥0.211).

### CA1 Area of the Hippocampus

In the CA1 area of the hippocampus, the tones evoked a two-peaked negative deflection, the first peak occurring at about 20 ms and the second at about 40 ms from stimulus onset (i.e., not from deviance which occurred at 25 ms after stimulus onset). This deflection had returned to or exceeded the baseline level by the end of the response time (Figure 3).

The LFPs were displaced towards negative polarity in response to the deviant tone relative to the standard tone in the SSI^500/^SDI^200^ condition (Figure 3) as reflected by a significant stimulus×time-segment -interaction, F(2,14) = 11.34, P = 0.010. Post-hoc pairwise comparisons further indicated that, in this condition, the displacement was present (P<0.05) in the first time segment (51.5–89 ms from duration change, P<0.001) and in the second time segment (89.5–127 ms from duration change, P<0.05). In the other conditions, no significant stimulus×time-segment interactions (P≥0.202) were observed.

For the SSI^500/^SDI^200^ condition, a main effect of stimulus, F(2,14) = 5.870, P = 0.046 and for the SSI^200/^SDI^200^ condition, a trend towards this effect, F(2,14) = 3.874, P = 0.090, were also observed. This effect was not present (P≥0.440) in the remaining two conditions.

### DG of the Hippocampus

In the DG area of the hippocampus, the general waveform of the LFPs to the tones themselves closely resembled those observed for the CA1 area. Again, in the averaged waveform, a negative deflection with slight peaks at about 20 ms and 40 ms from stimulus onset (that is, not from duration change) and a gradual return to the baseline level by the end of the response time were observed (Figure 3).

Furthermore, just as in the CA1, the LFPs in the DG area were displaced towards negative polarity in response to the deviant tone relative to the standard tone in the SSI^500/^SDI^200^ condition (Figure 3), as reflected by a significant stimulus×time-segment -interaction in the SSI^500/^SDI^200^ condition, F(2,10) = 7.50, P = 0.030. Post-hoc pairwise comparisons further indicated that in this condition the displacement was present (P<0.05) in the first (51.5–89 ms from change) time segment (Figure 3).

For the SSI^200/^SDI^500^ condition, there was a significant stimulus×time-segment interaction, F(2,10) = 11.362, P = 0.015. However, post-hoc comparisons only showed a trend for the deviant-standard difference in the last (127.5 ms −165 ms) time segment (P = 0.09). For the SSI^200/^SDI^200^ condition, a trend towards such an interaction (P = 0.085) was present, and for the SSI^500/^SDI^500^ condition, no such interaction was observed (P = 0.772).

No main effect of stimulus was present in any condition (P≥0.279).

### Subiculum

In the subiculum, the general waveform of the LFPs to the tones themselves was similar to those observed for the CA1 and DG areas. Again, in the averaged waveform, a negative deflection with slight peaks at about 20 and 40 ms from stimulus onset (that is, not from duration change) had returned to the baseline level by the end of the response time (Figure 3).

Furthermore, as in the CA1 and DG, the LFPs in the subiculum were displaced towards negative polarity in response to the deviant tone relative to the standard tone, as reflected by a significant stimulus×time-segment -interaction, F(2,16) = 8.18, P = 0.017, in the SSI^500/^SDI^200^ condition. Post-hoc pairwise comparisons further indicated that in this condition, the displacement was present (P<0.05) in the first (51.5–89 ms from change) time segment (Figure 3). No significant stimulus×time-segment interaction was observed in other conditions (P≥0.264).

There was only a trend for the main effect of stimulus (P = 0.083) in the SSI^500/^SDI^200^ condition.

## Discussion

We found differential LFPs (mismatch responses) to duration deviant tones in the auditory cortex and in the hippocampal system (CA1, dentate gyrus, and subiculum) in urethane-anesthetized rats. The responses - positive polarity in the auditory cortical and negative polarity in the hippocampal sites - appeared in the same latency range (between 51.5 and 89 ms post-change) and in all the recording sites, but only in the SSI^500^/SDI^200^-condition. In the remaining three conditions, the findings were negative.

The polarity and latency range of auditory cortical mismatch responses in the SSI^500^/SDI^200^-condition correspond to previous findings in urethane anesthetized rats [18], [19], [22], [23]. There are three possible reasons for the absence of mismatch responses in the SSI^200^/SDI^500^-condition. First, the short inter-deviant interval (due to the 200-ms SSI) might have diminished mismatch responses by the fast-paced activation of their neural generator (in humans [43]–[45], in cats [15]). Secondly, the long (500-ms) SDI might have led to a high degree of degradation of the memory trace of the standard tone, preventing the detection of the deviant tone as not matching this trace [1], [7]. Third, the long SDI relative to the SSI might have been a less efficient additional temporal cue (inter-stimulus interval) for the detection of the (stimulus duration) deviant tone than the short SDI relative to the SSI [46].

In the SSI^200^/SDI^200^- and SSI^500^/SDI^500^-conditions, the stimulus assignments (the 25-ms tone as the deviant tone and the 75-ms tone as the standard tone, but not vice versa) and numbers (a half) of sweeps per averaged waveform were different from those in the SSI^500^/SDI^200^- and SSI^200^/SDI^500^-conditions. Therefore, why mismatch responses were absent precisely in the SSI^200^/SDI^200^- and SSI^500^/SDI^500^-conditions remains to be accounted for. However, some tentative explanations can be offered.

In the SSI^200^/SDI^200^-condition, the short inter-deviant interval (due to the 200-ms SSI) might have diminished the amplitude of mismatch responses through the fast-paced activation of their neural generator [15], [43]–[45] while the short decay time of the memory trace of the standard tone to be compared to the sensory input by the deviant tone (due to the 200-ms SDI, [1], [7]) might not have been sufficient to compensate for this diminution. In the SSI^500^/SDI^500^-condition, the decay time (the 500-ms SDI) might, in turn, have been too long as mismatch responses were observed in the SSI^500^/SDI^200^-condition (with the same SSI but a shorter SDI).

Mismatch responses in rats have been found to be higher in amplitude to deviant tones as lengthenings than shortenings of standard tones [21]. Therefore, mismatch responses in the SSI500/SDI200-condition and a trend for a late differential response in the DG area in the SSI200/SDI500-condition may have reflected the fact that these conditions also involved the deviant tone as a lengthening of the standard tone as opposed to the SSI200/SDI200- and SSI500/SDI500-conditions which only included the deviant tone as a shortening of the standard tone. The present pattern of observations may also reflect the fact that the deviant tone was distinguishable from the standard tone by two auditory features (tone duration and inter-stimulus interval) in the former pair of conditions as opposed to solely one feature (tone duration) in the latter pair [1], [47].

Most importantly, hippocampal and auditory cortical mismatch responses were found in the same latency range and in the same stimulus condition. This correspondence suggests that the hippocampal responses reflect the initial detection of deviant tones similarly to auditory cortical mismatch responses. It remains to be resolved whether these responses of different anatomical origins recruit a single but spatially distributed neural mechanism, spatially separate and independent neural mechanisms, or spatially separate but interconnected neural mechanisms. The nature of the mechanism(s) should also be further explored. Namely, the mechanisms could, similarly to those for MMN in humans [1], [8], have treated the deviant tone as a change in the standard tone. Auditory cortical mismatch responses in urethane-anesthetized rats [18], [19] and hippocampal mismatch responses in awake rabbits [16] to frequency deviant tones have been found to reflect such mechanisms. Nevertheless, the rarity of the deviant tone as such [26], [27] also remains a possible explanation for our findings. Future studies with appropriate control procedures, such as deviant-alone (e.g. [18]) or equal-probability (e.g. [19]) stimulus conditions, are needed to test the validity of the change-detection account. There is also need for deeper understanding of the registration and transient memory storage of repetitive standard tones at the neural level [1], [7]. Stimulus-specific adaptation, a decrease in neural responses to a repeated tone which is specific to the physical features of this tone, observed in different stages of the auditory pathway [48]–[51] as functionally distinct from auditory cortical mismatch responses [52], [53] could provide a useful neurophysiological tool to increase this understanding.

To summarize, the brains of urethane-anesthetized rats were found to generate mismatch responses to duration deviants with the same set of stimulus parameters and in the same latency range in the auditory cortex and in three locations in the hippocampal system (CA1, dentate gyrus, subiculum). The findings prompt the question whether the hippocampal system plays an active role in the auditory cortical manifestation of these responses and hence also possibly of MMN in humans.

## Materials and Methods

### Ethics Statement

The experiments were approved by the Finnish National Animal Experiment Board (Permit code: ESLH-2007-00662), and carried out in accordance with the European Communities Council Directive (86/609/EEC) regarding the care and use of animals for experimental procedures.

### Animals and Surgery

Ten Spraque Dawley rats were used in the experiment (weight 305–375 g). The animals were housed as groups in cages with water and feed ad libitum. The animals were anaesthetized with urethane (Sigma-Aldrich, St. Louis, MO, USA) i.p. (1.2 g/kg). The level of anesthesia was controlled by pedal withdrawal reflex, and if necessary, extra doses of urethane were given. The animal was positioned in a streotaxic instrument with blunt ear bars which afterwards were removed to allow auditory stimulation. Under lidocaine anesthesia (Lidocain 20%, Orion Pharma, Espoo, Finland) skin and muscle tissue were removed to expose unilaterally a 2×2 mm region over the left primary auditory cortex (from bregma anterior posterior (AP): −4.5– (−6.5) mm, dorsoventral (DV) 3–5 mm lateral to the bone edge of the upper skull surface).

A tip of a Teflon-insulated stainless steel wire (diameter 200 µm, A-M Systems, Carlsberg, WA, USA) was positioned on the surface of the dura above the auditory cortex on the basis of on-line recorded potentials. In addition, intracranial electrodes were implanted (Formwar® insulated stainless steel wire, diameter 100 µm, California Fine Wire Company Co, Grover Beach, CA, USA). Two electrodes with 400-µm tip separation were lowered to the ventral subiculum to coordinates AP: −6.5 mm, ML: 5.5 mm and DV 6.6. Three intermediate (caudal) hippocampal electrodes with 0.6 mm spacing between electrodes were lowered to coordinates AP: 6.0 mm, ML: 4.8, 5.4 and 6.0 mm, and DV: 4.6 mm. Based on histology, electrodes located in the area of interest were selected for further analysis. For the reference electrode, a hole was drilled in the scull over the right side of the cerebellum and a small insulin needle (BD Lo-Dose syringe, USA) was inserted in the cerebellum (AP −10 mm, ML: 2–3 mm and DV: 2 mm) and the animal was grounded by inserting a needle (18G, Terumo, Somerset, NJ, USA) subcutaneously into the neck.

### Stimuli and Procedure

Sinusoidal 4000-Hz tones of 25 ms and 75 ms in duration, including 5-ms rise and fall times, were used as stimuli. The sound pressure level for each tone was 70 dB with C-weighting (optimized for 40–100 dB measurement), as measured with a sound level meter (type 2235, Bruel & Kjaer, Nærum Denmark), in the location of the animal’s right pinna during the recording.

The deviant (P = 0.1) and the standard (P = 0.9) occurred in a series of 1000 tones randomly with the restriction that consecutive deviants were separated by at least two standards.

Figure 2 illustrates the four stimulus conditions used in the study.

In two of the stimulus conditions, (SSI^200^/SDI^200^ and SSI^500^/SDI^500^), the SSI and the SDI was either 200 ms or 500 ms, respectively, and the deviant always 25 ms and standard 75 ms in duration. Each of these conditions comprised one stimulus series.

In the other two stimulus conditions (SSI^500^/SDI^200^ and SSI^200^/SDI^500^), the SSI and the SDI differed. In the SSI^500^/SDI^200^-condition, the SSI was 500 ms and the SDI 200 ms. In the and SSI^200^/SDI^500^-condition, SSI was 200 ms and SDI 500 ms. Each of these conditions comprised two stimulus series to counterbalance the stimulus assignments (both durations assigned to both stimulus types across the series).

The sequential order of the six series (one per SSI^200^/SDI^200^- and SSI^500^/SDI^500^-conditions, respectively, and two per SSI^200^/SDI^500^- and SSI^500^/SDI^200^- conditions, respectively) was counterbalanced across the animals to control for effects of the duration of stimulus exposure and of the passage of time itself (e.g., through variations in the level of anesthesia).

The stimulus presentation was controlled by E-prime software (Pittsburg, PA, USA), and the stimuli were delivered from a PC via an active loudspeaker system (Studiopro 3, M-audio, Irwindale, CA, USA). The stimulation was presented via a passive loudspeaker directed towards the right ear of the animal at a distance of 20 cm.

### Electrocortical and Hippocampal Recordings

After surgery, the right ear bar was removed and recording started. Continuous electrocorticogram and hippocampal EEG were first 10-fold amplified using the MPA8I preamplifier (Multichannelsystems, Reutlingen, Germany), high-pass filtered at 0.1 Hz, 50-fold amplified, and low-pass filtered at 5000 Hz using an FA32I filter amplifier (Multichannelsystems), low-pass filtered at 400 Hz using a CyberAmp 380 filter amplifier (Molecular Devices Corporation), and finally sampled with 16-bit precision at 2 kHz (DigiData 1320A, Molecular Devices Corporation). The data were stored on a computer hard disk using Axoscope 9.0 data acquisition software (Molecular Devices Corporation).

### Off-line Analysis

The data analyses were performed offline using a Vision Analyzer (Brain Products, Gilching, Germany), Matlab 7.5 (MathWorks Inc., Natick, MA, USA). For the hippocampal recordings, the electrodes successfully implanted in the targeted areas were applied in the analysis (9 animals for the subiculum, 8 for the CA1 area, and 6 for the dentate gyrus, Figure 1).

First, artifacts were removed from the data. Electrocardiogram epochs containing voltage steps larger than 300 µV/ms were deleted for 200 ms before and after the artifact. Visual inspection of the data revealed that no other types of artifacts were present in the data.

The data were then offline-filtered (0.1–30 Hz, 24 dB/octave, Butterworth Zero Phase filters), segmented (for each deviant and its immediately preceding standard), and baseline corrected against the mean activity in the ±25 ms from tone onset and, hence in the 50-ms time window prior to duration deviance.

Finally, the artifact-free segments were averaged for each animal separately for deviants and immediately preceding standards for each condition. The averaging was also made across two stimulus blocks (with the opposite stimulus assignments) in the SSI500/SSD200 and SSI200/SSD500 conditions and for one stimulus block in the SSI200/SSD200 and SSI500/SSD500 conditions. Not less than 67 out of 100 sweeps were included in calculating the average values per stimulus type and condition in any animal, the average number being 98.7 out of 100 sweeps. For the response amplitude analysis, the response time of 187.5 ms, as calculated from tone onset, was divided into five 37.5-ms segments. The last three of these segments (starting at 50 ms from change) which coincided with mismatch-like waveforms observable in the signal (see also previous findings of mismatch responses commencing at about 60 ms post-stimulus in urethane anesthetized rats [18], [19]) were included in the statistical analyses.

### Histology

After recording, the tips of the intracranial electrodes were marked in the tissue by anodal 30-µA 5-s current. The animal was sacrificed by cervical dislocation and the brain was moved from the skull and left for immersion post-fixation for 4 h in 4% paraformaldehyde (PFA) solution and after that in 30% sucrose solution for two days. The brains were stored in −20°C until slicing. Coronal sections (thickness 35 µm) were cut with a freezing slide microtome. The electrode locations were verified from the sections by cresyl violet staining and the exact locations of the electrode tips were confirmed by microscope observation.

### Statistical Analysis

The statistical analyses were performed with SPSS for Windows (SPSS Inc., Chicago, IL, USA). As there were no suitable non-parametric alternatives, ANOVA for repeated measures with stimulus (standard, deviant) and time-segment (first, second, third) as factors was used to justify post-hoc deviant-standard LFP amplitude comparisons for each time segment separately. Degrees of freedom were Greenhouse-Geisser corrected whenever the sphericity assumption was violated, and corrected P values were reported. Pairwise deviant-standard comparison for each time segment was made using the paired t test (two-tailed). An alpha level of 0.05 was used in all analyses.
